# Comparison of Outcomes between Two Methods to Extract Stone Fragments during Flexible Ureteroscopic Lithotripsy

**DOI:** 10.1155/2018/4526721

**Published:** 2018-05-30

**Authors:** Tadashi Tabei, Hiroki Ito, Kazuki Kobayashi, Takashi Kawahara, Junichi Matsuzaki

**Affiliations:** ^1^Department of Urology, Yokosuka Kyosai Hospital, 1-16 Yonegahama-Dori, Yokosuka, Kanagawa, Japan; ^2^Department of Urology, Yokohama City University Graduate School of Medicine, 3-9 Fukuura, Kanazawa-ku, Yokohama, Kanagawa, Japan; ^3^Departments of Urology and Renal Transplantation, Yokohama City University Medical Center, 4-57 Urafune-Cho, Minami-ku, Yokohama, Kanagawa, Japan; ^4^Department of Urology, Ohguchi Higashi General Hospital, 2-19-1 Irie, Kanagawa-ku, Yokohama, Kanagawa, Japan

## Abstract

**Objectives:**

To retrospectively compare the operative and clinical outcomes of flexible ureteroscopic lithotripsy (fURSL) with stone extraction performed either by a surgeon (SE) who manipulates the retrieval basket or by having the surgical assistant (AE) manipulate the retrieval basket with the aim of clarifying which method provides a greater stone-free postoperative status.

**Methods:**

The study group consisted of patients who underwent fURSL with SE or AE at our institution between April 2015 and December 2016. Demographic, clinical, stone, and operative variables were compared between the two groups. Multivariate logistic regression was used to identify risk factors associated with a stone-free and non-stone-free status postoperatively.

**Results:**

Our analysis included 196 cases of renal stones treated using fURSL, with 109 who underwent AE and 87 who underwent SE. The rate of stone-free status was higher for the SE group (90.8%) than for the AE group (61.5%; *P* < 0.001). The method of extraction was identified as an independent predictor of stone-free status (*P* < 0.001, odds ratio (SE compared to AE), 9.133, 95% confidence interval, 3.736–22.322).

**Conclusion:**

The stone-free rate is improved by having the surgeon perform the stone extraction as part of the fURSL procedure.

## 1. Introduction

With recent improvements in the design and use of endoscopes and peripheral devices, flexible ureteroscopic lithotripsy (fURSL) has become a safe and effective treatment for upper urinary tract lithiasis, achieving a higher stone-free rate than with shockwave lithotripsy (SWL) [1]. In fact, fURSL has become an alternative to percutaneous nephrolithotomy (PCNL), even for the removal of large renal stones [2, 3]. Reflecting this advancement in fURSL, the European Association of Urology (EAU) and the American Urological Association have both recommended the use of fURSL for various cases of upper urinary tract lithiasis [1, 4, 5].

The EAU guidelines on urolithiasis state that a dust-and-go strategy can be used for the treatment of large stones. In these cases, the dust-and-go strategy is considered to be effective despite the likelihood of residual fragments [6]. However, residual fragments are a potential cause of postoperative complications, including urinary tract infection and pain, as well as being a source of recurrent stone formation [7]. Therefore, complete extraction of stone fragments is considered to be essential for a successful procedure, lowering the risk for postoperative complications.

The procedures of fURSL can generally be divided into two phases, namely, stone fragmentation and stone extraction. Tipless nitinol baskets are the most popular tool for extracting stone fragments in fURSL due to their flexibility and handleability, which enables urologists to extract stones located in any of the renal calices. To the best of our knowledge, however, there is currently no consensus regarding “who should manipulate the basket” during surgery, the surgical assistant (assistant extraction, AE), or the operating surgeon (surgeon extraction, SE). In the absence of research evidence, the decision to proceed with AE or SE will be based on a surgeon's preference and is likely to vary widely between institutions and geographical regions and countries.

In our institution, we performed AE during fURSL until March 2016. As the SE method was adopted in the highest volume center performing fURSL in our country, it was more common in our region for the SE to perform the procedure rather than the AE. We have systematically adopted the SE method since April 2016. Therefore, our aim in this study was to retrospectively compare the operative and clinical outcomes of AE and SE in order to clarify which method provides a greater stone-free postoperative status.

## 2. Materials and Methods

The clinical criteria for the selection of SWL, PCNL, and fURSL for the treatment of urinary stones at our institution, Yokosuka Kyosai Hospital, are as follows. For urinary stones <20 mm in diameter, we usually recommend SWL and fURSL. For renal stones > 20 mm in diameter, PCNL is offered as the first-line treatment, with fURSL offered as the second choice. The final selection of the treatment modality is based on the patient's preference.

Between April 2015 and December 2016, we performed 220 fURSL procedures for renal stones. Of these 220 procedures, 24 cases were excluded from our analysis because of the following reasons: incomplete data on operative procedures and outcomes; more than two stages of fURSL performed; and bilateral procedure performed in the same operative session. Therefore, the data from 196 fURSL procedures were included in our analysis for evaluating the association between the method of extraction (AE or SE) and the rate of stone-free status (SFR) in a unilateral single procedure.

The following data were extracted from the medical records to identify factors that may influence the SFR for the two extraction methods: demographic factors (age, sex, height, and body mass index (BMI)); clinical factors (presence of hydronephrosis, presence of lower pole calculi, number of calyx-involving stones, and maximum and mean Hounsfield units (HUs)); stone-related factors (cumulative stone diameter (CSD), stone volume (SV), stone number, stone side, and stone composition); and operative factors (operative time, preoperative stenting, experience of the surgeon, method of stone extraction, and diameter of ureteral access sheath; length of postoperative stay and presence of postoperative fever > 38.0°C). The CSD was calculated as the sum of the maximum diameter of the stones measured on plain radiography of the kidneys, ureters, and bladder (KUB images). SV was calculated using the ellipsoid formula as follows: height (mm) × width (mm) × depth (mm) × *π*/6, with measurements obtained from preoperative noncontrast computed tomography (NCCT). Stone-free status, the primary outcome, was defined as the absence of visible fragments on KUB films obtained 2–4 weeks after stent removal. If a postoperative ureteral stent was not placed, the stone-free status on KUB film was assessed 2–4 weeks after fURSL. Cases of nonopaque stones were defined by the absence of visible fragments on NCCT. All KUB films or NCCTs were assessed by a single urologist (TT).

The methods used in the present study were approved by our institutional review board.

### 2.1. Surgical Techniques

We have previously described our surgical procedures [8], with relevant features summarized as follows. Preoperative urine cultures were performed for all patients. Patients were administered intravenous antibiotic prophylaxis from time of initiation of anesthesia until postoperative day 1. If the urine culture was negative, cefazolin (2 g/day) was used. For patients with a positive urine culture, the appropriate antibiotics were selected based on the culture.

All 196 procedures were performed under general anesthesia and in the lithotomy position. We first performed a cystoscopy to exclude intravesical lesions. Following confirmation, a 6/7.5 Fr semirigid ureteroscope (Wolf™, Knittlingen, Germany) was inserted into the ureteropelvic junction and a guidewire was place in the true lumen of the ureter. Ureteral access sheaths (Navigator™ 11/13 or 12/14 or 13/15 Fr, Boston Scientific, Natick, MA) were placed through the guidewire in all procedures to facilitate stone extraction and to reduce intrarenal pressure. The irrigation flow was adjusted manually by an assistant. A flexible ureteroscope (Olympus V2™ or P-5™, Olympus, Tokyo, Japan) was used to fragment the stones with a 200 *μ*m Holmium: yttrium–aluminum–garnet laser (Versa Pulse 30 W; Lumenis, San Jose, CA, USA). Lower pole calculi were repositioned before fragmentation. For stone removal and clearance of residual fragments, 1.5 Fr tipless nitinol baskets (NCircle; Cook Medical) were used in all cases. A ureteral stent was placed postoperatively if one of the following conditions was met: presence of residual stones, likelihood of a second-look procedure, operative time > 60 min, presence of ureteral wall injury, and solitary kidney. Postoperative stenting was also performed at the surgeon's discretion. A 14 Fr conclusion urethral balloon catheter was placed for all cases.

Procedures from April 2015 to March 2016 were performed using AE, while cases performed from April 2016 to December 2016 were performed using SE, with the basket attached to the ureteroscope by a basket holder (M-Arm™, MC medical, Tokyo, Japan). Switching to AE method was permitted if the surgeon had difficulty in continuing with the SE method.

### 2.2. Statistical Analysis

Measured demographic, clinical, stone-related, and operative variables were compared between the AE and SE groups. Continuous variables were expressed as a median (minimum- maximum), with between-group differences evaluated using a Mann–Whitney *U* test. Between-group differences for categorical variables were evaluated using chi-squared analysis. The cutoff SV and CSD values for stone-free status were identified using a receiver operating characteristic (ROC) curve analysis, with a follow-up multivariate logistic regression to identify risk factors predictive of a stone-free status. The level of significance was set at a *P* value < 0.05, and all analyses were performed using SPSS (version 19, Chicago, IL, United States).

## 3. Result

Of the 196 cases of fURSL included in our analysis, 109 were performed with AE and 87 with SE. There were no cases in which surgeons switched from the SE to the AE method for stone extraction. Measured demographic, clinical, stone, and operative variables are summarized in Table 1 for the SE and AE groups. Variables were comparable between the two groups, except for the following. SV and CSD tended to be larger in the SE group than in AE group, although this difference was not significant (*P* = 0.178 and 0.051, respectively). The maximum HUs were higher in the SE group than in the AE group (*P* = 0.035). Surgeons preferred using a thinner ureteral access sheath for the SE procedure compared with the AE procedure (*P* = 0.001). The stone-free rate was higher for the SE group than for the AE group (90.8% versus 61.5%, respectively, *P* < 0.001), despite there being no difference in operative time between the two groups. Postoperative length of stay and the incidence rate of a postoperative fever were comparable for both extraction methods.

**Figure 1:**
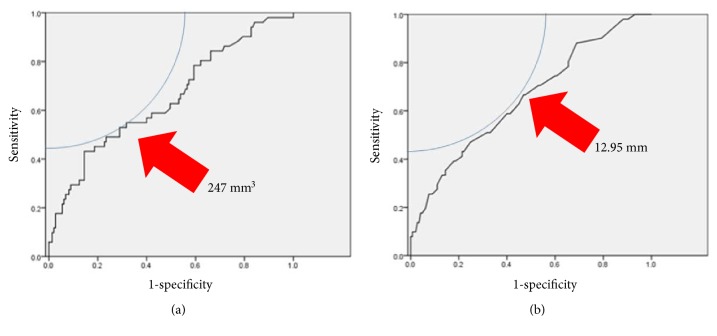
ROC curve showing the cutoff stone volume (a) and cumulative stone diameter (b) values for a stone-free status.

We also compared demographic, clinical, stone, and operative variables between patients of postoperative stone-free (SF) or non-stone-free (NSF) status. Between-group differences were identified in the number of stones (1.0 versus 2.0, SF and NSF respectively, *P* = 0.006), number of involved calyces (0 versus 1.0, *P* = 0.010), SV (190 mm^3^ versus 382 mm^3^, *P* = 0.002), CSD (10.0 mm versus 14.0 mm, *P* = 0.001), and extraction method (SE: 54% versus 16%, *P* < 0.001). All other variables were comparable between the two groups, including the rate of preoperative stenting, lower pole calculi and hydronephrosis, and HUs, as well as the experience of the operator. The ROC curves identifying the SV and CSD cutoff values associated with a stone-free status are shown in Figure 1. The cutoff value of SV was 247 mm^3^ and 12.95 mm for CSD. On multivariate logistic regression analysis, the method of stone extraction was identified as an independent factor of a stone-free postoperative status (*P* < 0.001, odds ratio (SE compared to AE), 9.133, 95% confidence interval, 3.736 to 22.322; Table 2).

A comparison of measured demographic, clinical, stone, and operative variables between patients* with* and* without *a stone-free status for the AE and SE methods is presented in Tables 3 and 4, respectively. For the AE method, several factors were predictive of a stone-free status (Table 3): age (*P* = 0.013), number of stones (*P* = 0.021), SV (*P* < 0.001), CSD (*P* < 0.001), and number of calices involved (*P* = 0.027). In contrast, lower pole calculi were the only significant factor (*P* = 0.002) predictive of a stone-free status for the SE method (Table 4). Operator experience was not a significant factor for either extraction method.

## 4. Discussion

We compared the effect of using AE or SE on the SFR among patients with renal stones treated using fURSL, providing evidence of a significant benefit of the SE method. In particular, compared to AE, SE was associated with a 9.133-fold increase in SFR. To the best of our knowledge, this is the first study that compared the effectiveness of AE and SE. Previous studies have reported the presence of lower pole calculi as the principal predictive factor for failure to achieve a stone-free status with fURSL [3, 9] because of the difficulty in accessing the stone with a ureteroscope, particularly when the angle between the axis of the inferior calyx and the ureteropelvic junction is steep [10]. The dimension of the stone is also an important factor to consider. Although the use of different parameters of stone size, including SV, surface area, and CSD, has limited our understanding of the direct effects of stone size on the SFR, it is generally accepted that multiple fURSL treatments are likely to be needed for renal stones < 20 mm in diameter [11]. Lim et al. reported a cumulative stone burden > 150 mm^2^ to be a significant predictor of residual fragments when using fURSL [9], where stone burden was calculated by multiplying the longest diameter by the perpendicular diameter of the stone to obtain a two-dimensional area. In a previous study, we reported that a stone-free status after fURSL could be predicted by SV, the presence of lower pole calculi, an operator's experience, the number of stones, and presence of hydronephrosis [12]. All data for that study were acquired from fURSL performed with SE.

In the present study, only the presence of lower pole calculi was found to be predictive of a stone-free status with SE. This difference, compared to our previously reported findings [12], is likely explained by the different methods used to assess stone-free status, namely, NCCT in our previous study and KUB film in our current study. We noted that NCCT provided a higher sensitivity for detecting residual fragments than all other imaging modalities [13]. The use of KUB film may have resulted in an overestimation of stone-free status and, hence, differences in identified predictive factors of the SFR. Furthermore, differences in patient characteristics between the studies might have influenced differences in findings. Our previous analysis was based on data obtained from the highest volume center performing fURSL procedures in Japan. By contrast, only cases from our institution were included in our current analysis, and the SFR was higher and SV smaller than among patients in our previous study. In fact, in a previous study, we identified a SV > 500 mm^3^ to be a predictive factor of a non-stone-free status with SE.

In the AE group, we identified SV, CSD, number of stones, and number of calices involved as risk factors of residual fragments. Since large volume stones and multiple stones are likely to bear more fragments, inevitably, a larger number of extraction procedures are required. As effective extraction requires a coordination between the timing of extraction procedures between the surgeon and the assistant for the AE method, the difficulty for AE will increase for large or multiple stones. By comparison, large or multiple stones did not influence the SFR when using the SE method. Therefore, for the range of stone sizes in our current study (0.17 to 4404.0 mL), SE was a more effective extraction method than AE to achieve a stone-free status, allowing successful extraction of larger stones than with AE. If an assistant fails to basket a stone appropriately, additional time would be needed to basket the stone to ensure complete stone retrieval. This increase in operative time likely explains the lower stone-free status obtained with AE than with SE, with a previous study having an upper limit of operative time of 90–120 min to avoid severe complications [14]. In our institution, this upper limit is not strictly adhered to, with some procedures exceeding the 120 min limit to ensure appropriate stone removal. From our analysis, we considered that the higher speed of stone extraction for the SE than AE method contributed to better stone-free status for the SE method, rather than differences in operative time.

As the use of SE in our institution is relatively new, we expected that experience will have an effect on the achievement of SFR. However, experience was not identified as a significant factor in the present study. The technical experience of assistants was not considered in our analysis. Certainly, the limited experience of assistants would have a large influence on the outcomes of the AE approach. However, we do not consider this factor as a significant influence in our study as all 5 assistants were urologists with prior experience in performing > 20 cases of fURSL to ensure a safe and effective operative procedure. It is important to note that lower pole calculi were predictive of a non-stone-free status with SE but not with AE. However careful attention should be paid while interpreting this finding. As shown in Tables 3 and 4, the SFR of the SE method for lower pole calculi is higher than that of the AE method (85% versus 50%). This finding indicates that the SE method can be influenced to a larger degree than the AE method because simultaneous manipulation of both the ureteroscope and the basket is more difficult in the lower calyx.

The limitations of our study need to be acknowledged. Foremost, our analysis was retrospective in nature, based on the data from a single-institution and included a relatively low number of cases (*n* = 196) with heterogeneity, with significant differences in stone composition and maximum HUs identified between the SE and AE groups. Of note, HU values were higher in the SE group than in the AE group, where higher HU values have been reported to be predictive of stone disintegration with SWL [15, 16]. Despite this unfavorable background for the SE group, the surgical outcomes of the SE group were superior to that of the AE group. Therefore, our conclusion was not affected by this significant difference in HUs. Third, we assessed the stone-free status using KUB films and not NCCT, despite the knowledge that NCCT provides a more sensitive assessment of the stone-free status. As cases from 14 surgeons were included in our analysis, the effects of heterogeneity on outcomes cannot be discounted.

In conclusion, the SFR can be improved by having a surgeon perform the stone extraction as part of the fURSL treatment. Our results should encourage the use of a single-surgeon approach to stone fragmentation and extraction for the treatment of renal stone using fURSL.

## 5. Conclusion

The stone-free rate is improved by having the surgeon perform the stone extraction as part of the fURSL procedure.

## Figures and Tables

**Table 1:** Comparison of measured demographic, clinical, stone, and operative variables between methods of extraction of stone fragments.

	AE	SE	*P* value
Number of patients	109	87	
Age, years	64 (20–90)	65 (24–89)	0.523
Sex			
Female	48 (44%)	45 (52%)	0.284
Male	61 (56%)	42 (48%)	
Side			
Right	52 (48%)	39 (49%)	0.597
Left	57 (52%)	48 (51%)	
Height (cm)	162 (139–180)	160 (128–181)	0.209
Body mass index (kg/m^2^)	23.9 (15.4–37.9)	24.0 (15.7–33.9)	0.407
Number of stones	1 (1–7)	1 (1–10)	0.201
Stone volume (mm^3^)	175.5 (0.175–4484)	249 (2.5–1830)	0.178
Cumulative stone diameter (mm)	10 (0.5–54)	13 (2–41)	0.051
Lower pole calculi (*n*)	40 (37%)	34 (39%)	0.491
Number of calyx involving stone			
0	59 (54%)	42 (48%)	0.716
1	34 (31%)	27 (31%)	
2	11 (10%)	13 (15%)	
3	5 (5%)	5 (6%)	
Hounsfield Units			
Maximum	1074 (152–1830)	1215 (284–1884)	0.035^*∗*^
Mean	751 (151–1671)	853 (134–1530)	0.554
Hydronephrosis (*n*)	32 (29%)	31 (36%)	0.35
Access sheath (Fr)			
13	9 (8%)	25 (29%)	0.001^*∗*^
14	35 (32%)	18 (21%)	
15	65 (59%)	44 (56%)	
Preoperative stenting (*n*)	67 (61%)	47 (54%)	0.294
SWL failure (*n*)	13 (12%)	8 (9%)	0.449
Operator's experience of fURSL	15 (1–50)	11 (1–156)	0.075
Operative time (min)	85 (18–194)	80 (22–149)	0.182
Postoperative length of stay	2 (1–9)	2 (0–7)	0.115
Postoperative fever (*n*)	20 (18%)	15 (17%)	0.306
Stone free (%)	61.5	90.8	<0.001^*∗*^
Stone composition			
CaOX	77 (71%)	34 (39%)	0.001%^*∗*^
CaP	3 (3%)	3 (3%)	
UA	2 (2%)	3 (3%)	
MAP	6 (6%)	14 (16%)	
Mixed	17 (16%)	29 (33%)	
other	1 (1%)	0 (0%)	
unknown	3 (3%)	4 (5%)	

AE: assistant extraction; SE: surgeon extraction; CaOX: calcium oxalate; CaP: calcium phosphate; UA: uric acid; MAP: magnesium ammonium phosphate; ^*∗*^statistically significant.

**Table 2:** Multivariate logistic regression analysis of risk factors for residual fragments after fURSL (*n* = 196).

		Multivariate		
		*P*	OR	95% CI
Number of stones	1	0.943	0.943	0.349–2.549
	≥2		1	
Number of involved calices	1	0.212	0.495	0.164–1.495
	≥2		1	
Stone volume (mm^3^)	<247	0.196	0.537	0.209–1.379
	≥247		1	
Cumulative stone diameter (mm)	<12.95	0.448	0.656	0.221–1.951
	≥12.95		1	
Lower pole calculi	absent	0.524	0.749	0.307–1.824
	present		1	
Extracting method	AE	<0.001^*∗*^	9.133	3.736–22.322
	SE		1	

AE: assistant extraction; SE: surgeon extraction; ^*∗*^statistically significant.

**Table 3:** Comparison of measured demographic, clinical, stone, and operative variables between patients with a ston-free and a non-stone-free postoperative status for the AE group.

	Non-stone free	Stone free	*P* value
Number of patients	43	66	
Age (years)	67 (46–90)	61.5 (20–90)	0.013^*∗*^
Sex			
Female	23 (53%)	25 (38%)	0.109
Male	20 (47%)	41 (62%)	
Side			
Right	21 (49%)	32 (48%)	0.971
Left	22 (51%)	34 (52%)	
Height (cm)	162 (139–180)	161 (139–180)	0.47
Body mass index (kg/m^2^)	23.3 (17.9–37.9)	24.4 (15.4–37.9)	0.401
Number of stones (n)	1 (1–7)	1 (1–3)	0.021^*∗*^
Stone volume (mm^3^)	175.5 (0.175–4484)	120 (1.0–1989)	<0.001^*∗*^
Cumulative stone diameter (mm)	13 (4–54)	8 (0.5–26)	<0.001^*∗*^
Lower pole calculi (*n*)	20 (47%)	20 (30%)	0.086
Number of calyx involving stone			
0	16 (37%)	43 (65%)	0.027^*∗*^
1	17 (40%)	16 (24%)	
2	6 (14%)	5 (8%)	
3	4 (9%)	2 (3%)	
Hounsfield Units			
Maximum	1179 (366–1830)	973 (152–1780)	0.061
Mean	858 (354–1671)	684 (151–1516)	0.059
Hydronephrosis (*n*)	12 (28%)	20 (30%)	0.788
Access sheath (Fr)			
13	3 (7%)	6 (9%)	0.845
14	15 (35%)	20 (30%)	
15	25 (58%)	40 (61%)	
Preoperative stenting (*n*)	30 (70%)	37 (56%)	0.151
SWL failure (*n*)	3 (7%)	10 (15%)	0.198
Operator's experience of fURSL	18 (1–50)	14 (2–40)	0.302
Stone composition			
CaOX	31 (72%)	46 (70%)	0.752
CaP	1 (2%)	2 (3%)	
UA	1 (2%)	1 (2%)	
MAP	4 (9%)	2 (3%)	
Mixed	5 (12%)	12 (18%)	
other	0 (0%)	1 (2%)	
unknown	1 (2%)	2 (3%)	

AE: assistant extraction; CaP: calcium phosphate UA: uric acid MAP: magnesium ammonium phosphate; ^*∗*^statistically significant.

**Table 4:** Comparison of measured demographic, clinical, stone, and operative variables between patients with a stone-free and a non-stone-free postoperative status for the SE group.

	Non-stone free	Stone free	*P* value
No. of patients	8	79	
Age (years)	56 (24–82)	65 (24–89)	0.16
Sex			
Female	3 (38%)	42 (53%)	0.398
Male	5 (63%)	37 (47%)	
Side			
Right	2 (33%)	37 (47%)	0.237
Left	6 (67%)	42 (53%)	
Height (cm)	170 (143–180)	160 (128–181)	0.095
Body mass index (kg/m^2^)	23.5 (17.4–26.5)	24.0 (15.7–33.9)	0.467
Number of stones (*n*)	2.5 (1–10)	1 (1–10)	0.082
Stone volume (mm^3^)	276 (123–735)	249 (2.47–1830)	0.78
Cumulative stone diameter (mm)	17.8 (7.8–26)	13 (2–41)	0.056
Lower pole calculi (*n*)	5 (63%)	29 (37%)	0.002^*^
Number of calyx involving stone			
0	2 (25%)	40 (51%)	0.061
1	2 (25%)	25 (32%)	
2	2 (25%)	11 (14%)	
3	2 (25%)	3 (4%)	
Hounsfield Units			
Maximum	1197 (510–1606)	1228 (284–1884)	0.994
Mean	899 (382–1251)	821 (134–1530)	0.681
Hydronephrosis (*n*)	1 (13%)	30 (38%)	0.152
Access sheath (Fr)			
13	1 (13%)	24 (30%)	0.567
14	2 (25%)	16 (20%)	
15	5 (63%)	39 (49%)	
Preoperative stenting (*n*)	3 (38%)	44 (56%)	0.325
SWL failure (*n*)	1 (13%)	7 (9%)	0.9
Operator's experience of fURSL	6 (1–155)	11 (1–156)	0.154
Stone composition			
CaOX	4 (50%)	30 (38%)	0.65
CaP	0 (0%)	3 (4%)	
UA	1 (13%)	2 (3%)	
MAP	1 (13%)	13 (16%)	
Mixed	2 (25%)	27 (34%)	
other	0 (0%)	0 (0%)	
unknown	0 (0%)	4 (5%)	

SE: surgeon extraction; CaP: calcium phosphate UA: uric acid MAP: magnesium ammonium phosphate; ^*∗*^statistically significant.

## Data Availability

The datasets used during the current study are available from the corresponding author on reasonable request.

## Abbreviation (alphabetical order)

CSD: Cumulative stone diameter
fURSL: Flexible ureteroscopic lithotripsy
HUs: Hounsfield units
NCCT: Noncontrast computed tomography
PCNL: Percutaneous nephrolithotomy
ROC: Receiver operating characteristic
SFR: Stone-free rate
SV: Stone volume
SWL: Shockwave lithotripsy.
